# Measures of knee internal and external rotation made with a digital inclinometer are consistent with the measures made with an electromagnetic tracking system

**DOI:** 10.1002/jeo2.70209

**Published:** 2025-03-11

**Authors:** Mark K. Timmons, Hunter G. Copley, Dustin T. Darnell, Seth T. Jude, Gary McIlvain

**Affiliations:** ^1^ School of Health and Movement Sciences, College of Health Professions, Marshall University Huntington West Virginia USA

**Keywords:** knee rotation, knee stability, medial collateral ligament

## Abstract

**Purpose:**

Individuals with excessive knee rotation have higher levels of dysfunction and disability. An inexpensive, and reliable method to assess knee internal (IR) and external (ER) rotation will improve the assessment of knee injuries. The study explored the reliability and measurement error of two methods of knee rotation measurement. The study tested the hypothesis that the digital inclinometer will provide reliable measures of knee internal and external rotation and that the digital inclinometer measurements will be consistent with the measurements made with the electromagnetic tracking system.

**Methods:**

Twenty (20) participants without knee injury participated. Knee IR and ER were measured using electromagnetic tracking and a digital inclinometer. The intraclass correlation coefficient (ICC_(2,1)_) was calculated for both techniques.

**Results:**

The within device ICC values ranged from 0.826 to 0.939 for both devices. The within‐device minimal detectable change (MDC) ranged from 1.2° to 1.9°. The ICC values for EI and IR measures collapsed between the devices, ranged from 0.717 to 0.859. The MDC calculated between devices ranged from 1.6° to 1.9°.

**Conclusion:**

The results of the current study show that knee IR and ER can be measured reliably with both measurement techniques. The measurement of knee ER and IR did not differ between the two devices or between the right and left sides.

**Level of Evidence:**

Level IV, diagnostic, case series study.

**Abbreviations**
AMRI: antero‐medial rotatory instability
dMCL: deep medial collateral ligament
ER: external rotation
ICC: intraclass correlation coefficient
IR: internal rotation
MCL: medial collateral ligament
MDC: minimal detectable change
SEM: standard error of the measure
sMCL: superficial medial collateral ligament

## INTRODUCTION

Ligamentous medial knee injuries are common and can lead to physical dysfunction and disability [10, 11, 23]. While most injuries of the medial knee ligaments heal with conservative treatment, some knee injuries leave the knee with antero‐medial rotatory instability (AMRI) and frequently require surgical intervention and extensive rehabilitation to restore knee function [11]. The medial collateral ligament (MCL) provides transverse plane (rotational) stability, particularly as the knee flexion angle increases [12, 26, 36]. The MCL has two major anatomical divisions, the superficial (sMCL) and deep fibres (dMCL). Disruption of the dMCL decreases the rotatory stability of the knee [29, 39]. Injury to the posterior oblique ligament (POL) and the anterior cruciate ligament (ACL) are also shown to lead to increased knee external rotation [5, 7]. Patients with knee injuries resulting in increased knee external rotation have been shown to have poorer prognosis then patients without increase knee external rotation [37]. Assessing a patient's knee rotation might help better direct their care leading to improved prognosis of patients with dMCL, POL or ACL injury or AMRI.

Many methods have been proposed to measure transverse plane knee rotation [20, 21]. Specifically, several papers describe devices and procedures to measure knee internal and external rotation [3, 4, 13, 16, 17, 18, 19, 22, 30, 33, 38]. These devices have been used to measure knee rotation in individuals with and without knee injury. Monaco et al. [19], Naendrup et al. [22], Kuroda et al. [13] and Hoshino et al. [9], described electromagnetic motion tracking to measures knee rotation and displacement during the pivot shift test. These methods have been shown to provide valid and reliable measures of knee rotation and displacement. However, the methods described require expensive equipment, technical experience and considerable time to complete the testing. Digital inclinometers have been shown to provide reliable measures of sagittal plane motion of the knee [1, 8]. The literature has not described a simple and reliable method to assess transverse plane knee rotation. An inexpensive, less complex and expedient method of measuring knee rotation will improve the clinical evaluation of the knee.

The current study explored the measurement reliability of knee internal and external rotation made with a digital inclinometer. The external validity of the knee rotation measurements made with the digital inclinometer was tested against the measurements of knee rotation made with an electromagnetic tracking system. The study tested the hypothesis that the digital inclinometer will provide reliable measures of knee internal and external rotation and that the digital inclinometer measurements will be consistent with the measurements made with the electromagnetic tracking system.

## METHODS

**Figure 1:**
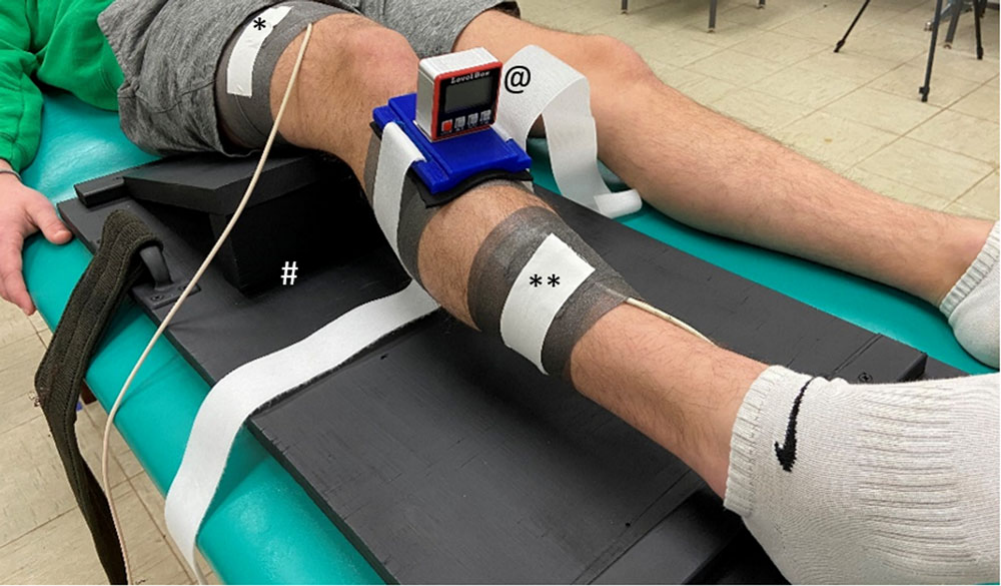
Digital Inclinometer and electromagnet sensor placements. *Proximal electromagnetic thigh sensor, **distal electromagnetic tribal senor, @digital inclinometer and #costume made knee support.

Twenty (20) participants (Table 1) without a history of a knee injury were recruited from a population of convenience for a repeated measures investigation. Participants were excluded if they reported pain in their lower extremities at the time of the testing or had a history of knee injury or had passive knee flexion less than 120°. All participants provided written informed consent prior to data collection. The investigation was approved by the University's Institutional Review Board (IRBnet #1958910‐1). All testing was completed during a single testing session. Passive knee internal and external rotation were measured using two methods concurrently. Method one, used a digital inclinometer (Figure 1) strapped to the participant's knee and the investigator moved the participant's knee into maximum internal and external rotation. In method two, investigators used electromagnetic tracking (Figure 1) to measure passive knee internal and external rotation. For both measurements the knee was flexed to 30°, the knee flexion angle was maintained by securing the test leg in a custom‐made apparatus (Figure 1). The 30° knee flexion angle was chosen because this knee flexion position is used in the assessment of the medial knee ligaments [6]. The measures of knee rotation by both measurement methods were made simultaneously. Three trials of both internal and external knee rotation were performed and the mean of the three measures was used for analysis.

**Table 1:** Participant demographics, mean ± standard deviation.

Gender	13 Female/7 male
Side dominance	20 Right
Age (years)	20.4 ± 1.1
Height (cm)	174.6 ± 11.1
Weight (kg)	87.1 ± 29.6
Lysholm Knee Score	98.9 ± 2.8

*Note*: Lysholm Knee Score 0–100, higher score indicates high function.

Knee external and internal rotation were measured using a digital inclinometer (Acemeter Technologies). A custom‐made jig held the digital inclinometer, the jig and inclinometer were positioned over the tibial crest and held in place with Velcro strapping (Figure 1). When the inclinometer was secured in place, the inclinometer was calibrated to read zero degrees. The investigator then moved the knee into maximal knee internal and external rotation. The maximal rotation angles were recorded to the nearest whole degree (°). After recording the knee rotation, the participants knee was returned to the resting position and the digital inclinometer was recalibrated to read zero degree. No measurement of the rotational torque was made, knee rotation was stopped when the examiner felt a firm end point. The same investigator performed all knee rotation measurements, the investigator is a licensed healthcare professional with more than 20 years of experience. The investigator would move the participants knee into either internal or external rotation until the investigator perceived great resistance to further rotation.

Three‐dimensional knee kinematics were measured using The Ascension TrackStar electromagnetic‐based motion capture system (Ascension Technology, Shelburne, VT) used with The Motion Monitor software (Innovative Sports Training, Inc, Chicago, IL). The current study used the electromagnetic tracking method described by Naendrup et al. [22] and Adler [2]. The electromagnetic sensors were attached to the participants' skin (Figure 1) with adhesive tape. One sensor was affixed to the participant's distal thigh. The second was affixed to the distal tibia. A third sensor was used as a digitising wand and was used to digitise bony landmarks while the participant is laying supine on an examination table. The thigh segment was identified proximally by digitising the anterior inferior iliac crest, and distally by the midpoint of the line between the lateral and medial femoral condyle. The tibial segment was identified by the midpoint of the line between the lateral and medial tibial condyles proximally and the midpoint of a line between the lateral and medial malleolus distally. Knee kinematics were collected concurrently with the digital inclinometer measures.

### Data analysis

Means and standard deviation for the maximal internal and external rotation were calculated. Differences in knee rotation measures were determined using paired T test, statistical differences were determined at *p* ≤ 0.05. Agreement within and between the two measurement techniques was determined using the interclass correlation coefficient model (2, 1) (ICC) [15, 28]. Agreement between measures at ICC values 0.81–1.00 are considered very good, good for 0.61–0.80, moderate for 0.41–0.60, fair for 0.21–0.40, and poor for values below 0.20 [25]. Sample size estimation based on minimal acceptable ICC of 0.50, an expected ICC value of 0.80 and a statistical power set at 0.80 showed that a sample size of 18 participants was required [34]. The standard error of the measure (SEM) and minimal detectable change (MDC) for the ER and IR measures were calculated for each method of measurement. Measurement error was calculated with the standard error of measure SEM = standard deviation × [√(1 − ICC)], which estimates the error about a single measure of a variable. The MDC represents the error when a measure is taken twice (change over time) and will be calculated by multiplying the SEM by √2 [31, 35]. All statistical calculations were performed using SPSS 24.0 (IBM, Chicago, IL).

## RESULTS

Twenty (20) participants completed all testing, participant demographic can be found in Table 1. The within‐device ICC values ranged 0.826–0.939. The within‐device MDC ranged 1.2°–1.9°. Collapsing the results across the devices showed consistent measures for both ER and IR (Table 2). The ICC values for right side and left side ER and IR made on both devices ranged 0.717–0.859. The MDC calculated between devices ranged 1.6°–2.0°.

**Table 2:** Measures of knee internal and external rotation collapsed across devices.

	Mean	StD	ICC	SEM	MDC
Internal rotation					
Right	17.1	2.80	0.750	1.4	2.0
Left	15.9	2.69	0.717	1.4	2.0
External rotation					
Right	16.8	2.79	0.812	1.2	1.7
Left	17.8	2.93	0.859	1.1	1.6

Abbreviations: ICC, intraclass correlation coefficient; MDC, minimal detectable change; SEM, standard error of the measure; StD, standard deviation.

The measures of IR and ER were consistently greater when measured with the inclinometer than with the electromagnetic device (Table 3). But these differences either did not reach statical significance or were less than the MDC calculated in the current study. The difference between devices (Table 3) for IR did not reach a statistical difference on either right side (mean difference = 0.4° ± 0.57°, *t* = 0.710, *p* = 0.486) or left side (mean difference = 0.7° ± 0.56°, *t* = 0.1.284, *p* = 0.214). The difference between devices for right side ER did not reach a statistical difference (mean difference = 0.9° ± 0.47°, *t* = 1.85, *p* = 0.084). The difference between devices for left side ER did reach a statistical significance (mean difference = 1.0° ± 0.42°, *t* = 2.481, *p* = 0.023). This difference between devices on the left side ER needs to be interpreted with caution, because the left side difference ER between the devices is 1.0°, this value is less than the calculated SEM (1.1°) and MDC (1.6°) for ER.

**Table 3:** Measures of knee internal and external rotation by side and measurement device.

	Mean	StD	Difference	ICC	SEM	MDC
Internal rotation						
Right						
Digital inclinometer	17.3	3.2	0.4	0.871	1.2	1.6
Electromagnetic	16.9	3.2		0.826	1.3	1.9
Left						
Digital inclinometer	16.2	2.9	0.7	0.923	0.9	1.2
Electromagnetic	15.5	3.0		0.837	1.2	1.7
External rotation						
Right						
Digital inclinometer	17.4	3.3	0.08	0.928	0.9	1.3
Electromagnetic	16.6	2.9		0.923	0.8	1.3
Left						
Digital inclinometer	18.3	3.4	1.0	0.939	0.9	1.3
Electromagnetic	17.3	2.8		0.854	1.1	1.5

*Note*: Measures of rotation are presented in degrees.

Abbreviations: Difference, difference between the digital inclinometer and electromagnetic measures; ICC, intraclass correlation coefficient; MDC, minimal detectable change; SEM, standard error of the measure; StD, standard deviation.

## DISCUSSION

Reliable measurements of right and left knee IR and ER with the knee at 30° flexion were made using both measurement devices. The measurements made with the digital inclinometer were consistent with the measurements made with the electromagnetic tracking system. The results of the current study suggest that the digital inclinometer and electromagnet tracking techniques provide reliable measurements of knee rotation at 30° knee flexion. The digital inclinometer might provide a less complex and inexpensive method to measure knee rotation in the clinical setting. The 30° knee flexed position tested in the current investigation is close to the knee flexion angle at initial contact of gait and while landing from a jump [14].

The knee rotation measures in the current study are consistent with the knee rotation measures reported in the literature [17, 24, 32, 40]. The mean external rotation for both knees measured with the inclinometer was 17.9° ± 3.2° and internal rotation was 16.8° ± 2.8°. The total arc of motion was 34.7°. The differences between the current studies and prior studies are likely due to differences in study methods. Mihalinec et al. [18], reported smaller values for external rotation (mean = 15.7° ± 4.2°) and external rotation (mean = 13.6° ± 3.7°) in a larger sample size (73 participants) including older individuals without a history of knee injury. Tsai et al. [32], used a more complex method and measurement equipment. They did not report the end position of knee rotation but did report a total arc of motion in participants without knee injury. The age of the participants in the Tsai et al. [32], paper (mean age = 30.3 years) was close to the age of the participants of the current study and the total arc of motion (total arc of motion = 25.8° ± 5.9°, ICC 0.88 and SEM 1.8°). Comparing knee rotation using a wireless gyroscope and a rotary potentiometer methods Petrigliano et al. [24], reported a mean external knee rotation of 21.9° ± 1.2° and 22.2° ± 1.2° in cadaveric knees during a simulated pivot shift test. Cadaveric knees would not have muscle activity to resist the knee rotation, and the pivot shift test produces knee external rotation and valgus. The mean age of the cadaveric knees was 32.2 years greater than the age of the participants in the current study. A rotational force of 7 kg was applied, the rotational forces in the current study were not measured. Russel et al. [27] reported internal rotation of 12.6° ± 3.9° and external rotation of 13.5° ± 3.1° in cadaveric knees of much greater age (mean = 80.5 years). Mayer et al. [17], reported mean external rotation with the knee at 30° during a pivot shift test of 9.0 ± 6.6° in participants without knee injury. They reported external rotational force A 2 NM external rotation force was applied. The internal knee rotation measured in the current study is less than the knee rotation measured during normal gait. Zurcher et al. [40] reported peak internal knee rotation 11.8° ± 4.4° and 1.7° ± 1.8° during the stance phase of gait in participants without knee injury.

Martinez‐Cano et al. [16], reported a systematic review exploring the reliability of measurements of knee transverse plane rotation. Good to excellent intra‐rater and inter‐rater reliability of transverse plane knee rotation measurements in participants with and without knee injury was reported in 12 papers. Soude et al. [30] reported excellent intra‐rater reliability (ICC = 0.89) and inter‐rater reliability (ICC = 0.99) in 12 participants with ACL injury using a smartphone accelerometer. Mihalinec et al. [18], reported excellent intra‐rater reliability (ICC = 0.916–0.940) and good to excellent interrater reliability (ICC 0.822–0.933) using the DYNEELAX device in 73 participants without knee injury. The current study reports similar interrater reliability for the digital inclinometer and electromagnetic tracking devices for both internal and external knee rotation in 20 participants without knee injury.

The results of the current study are limited to the measurement of knee rotation with a digital inclinometer in individuals without a history of knee injury. The current study did not measure the knee rotation of individuals with a history of knee injury. Greater knee external rotation has been report in females compared to males and to be greater in younger compared to older individuals [4]. The current study did not test the reliability of the digital inclinometer measurements of knee rotation across sexes or age groups. Further work will be performed in patient populations to determine the clinical utility and predictive value of these measures. Future work will need to be performed to determine the reliability of the measurement of knee rotation with an inclinometer at knee flexion angles other than 30° flexion.

## CONCLUSION

The results of the current study show that knee ER and IR can be reliably measured using a digital inclinometer. Knee ER and IR measured with the digital inclinometer were consistent with the measurements made with an electromagnetic track system. These results indicate that an inexpensive digital inclinometer can be used to track the knee rotation in individuals without knee injury or AMRI. More work will need to be complete to determine if measurements of knee rotations made with digital inclinometers could aid in the treatment of patients with AMRI.

## AUTHOR CONTRIBUTIONS

All authors contributed to the study conception and design. Material preparation, data collection and analysis were performed by Mark Timmons, Hunter Copley, Dustin Darnell, Seth Jude, and Gary McIlvain. The first draft of the manuscript was written by Mark Timmons and all authors commented on previous versions of the manuscript. All authors read and approved the final manuscript.

## CONFLICT OF INTEREST STATEMENT

The authors declare no conflicts of interest.

## ETHICS STATEMENT

The investigation was approved by the University's Institutional Review Board (IRBnet #1958910‐1). Informed consent was obtained from all individual participants included in the study.

## Data Availability

The data that support the findings of this study are available from the corresponding author upon reasonable request.
